# Approach to the patient with acquired hypothalamic syndrome

**DOI:** 10.1210/clinem/dgag197

**Published:** 2026-05-11

**Authors:** Ellen K Grishman, Shana E McCormack

**Affiliations:** Division of Endocrinology, St. Jude Children's Research Hospital, Memphis, TN 38105, USA; Division of Endocrinology & Diabetes, Children's Hospital of Philadelphia, Philadelphia, PA 19104, USA; Department of Pediatrics, Perelman School of Medicine at the University of Pennsylvania, Philadelphia, PA 19104, USA

**Keywords:** hypothalamic syndrome, hypothalamic obesity, multiple pituitary hormone deficiencies, craniopharyngioma, suprasellar brain tumor

## Abstract

The hypothalamus integrates signals from the body and the environment and coordinates homeostatic responses in multiple physiologic processes, including water balance, stress response, temperature regulation, energy balance, feeding behavior, sleep-wake cycles, and reproduction. Endocrinologists care for individuals for whom hypothalamic injury, whether from tumors, their treatment, or other causes, impairs pituitary hormone production and secretion. The extent of other symptoms in affected individuals is also influenced the extent of hypothalamic injury. “Acquired hypothalamic syndrome” refers to this broader collection of problems attributable to hypothalamic injury, variably including not only pituitary hormone deficiencies, but also hypothalamic obesity (or rarely, diencephalic syndrome), sleep and circadian disruption, temperature dysregulation, and behavioral issues (including memory problems and impulse control). Our goal is to present a pragmatic guide to the multidisciplinary, collaborative evaluation and management of hypothalamic syndrome.

## Case presentation

**Figure 1:**
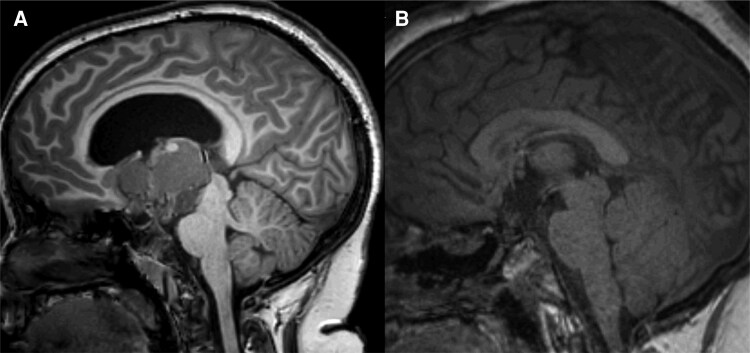
A, Case magnetic resonance imaging (MRI), pre procedure (left panel). A complex cystic and solid suprasellar mass is demonstrated, extending into and expanding the third ventricle. The mass contains central coarse calcifications, enhancing solid components, and numerous cysts of varying signal intensity. The mass measures approximately 4.7 × 4.2 × 4.6 cm (AP × TRV × CC). The optic chiasm is compressed and displaced anteriorly by the mass, with edema extending throughout the optic chiasm and into the prechiasmatic optic nerves. There is surrounding T2 hyperintensity extending into the hypothalamus and posterior limbs of the internal capsules bilaterally. The thalami and cerebral peduncles are splayed by the mass. No distant foci of nodularity or abnormal enhancement are identified. The cerebral sulci are diffusely small in size. The basal cisterns are narrowed but appear patent. There is no evidence of intracranial hemorrhage, restricted diffusion, or extra-axial fluid collection. The pituitary gland appears flattened along the floor of the sella, with nonvisualization of the posterior pituitary bright spot. B, Case MRI, post procedure (right panel). Prior transsphenoidal approach for resection of craniopharyngioma centered in the suprasellar cistern, with evolving postsurgical changes. Stable appearance of signal abnormality involving suprasellar and interpeduncular cisterns, inferior third ventricle, fornices, and anterior commissure, as described. Continued surveillance is recommended to ensure stability of these regions. No evidence of new or worsening parenchymal signal abnormality. Bifrontal encephalomalacia, particularly involving the parasagittal anterior frontal lobes. Bifrontal approach ventricular catheters, terminating in the frontal horns of the lateral ventricles, unchanged. Decompressed ventricular system, without evidence of hydrocephalus.

A 10-year-old child, assigned male sex at birth, presented to the emergency room with sudden onset of headache, facial droop, and unintelligible speech. Fatigue had been present for a few months. Brain magnetic resonance imaging (MRI) was obtained (Fig. 1A). The child underwent right frontal stereotactic guided biopsy with cyst fenestration; pathology was consistent with an adamantinomatous craniopharyngioma. Multiple pituitary hormone deficiencies were diagnosed, including arginine vasopressin deficiency (AVP-D, formerly called central diabetes insipidus) without intact thirst, central adrenal insufficiency, central hypothyroidism, and growth hormone (GH) deficiency. The patient ultimately underwent endoscopic endonasal tumor resection (Fig. 1B shows the postprocedure MRI). Obesity was present at tumor diagnosis, and body mass index (BMI) increased from approximately 132% of the 95th percentile to approximately 148% of the 95th percentile in the first approximately 9 months after definitive tumor treatment. Additional complications were diagnosed, including visual field deficits, metabolic-associated steatotic liver disease, obstructive sleep apnea, heat and cold intolerance, memory deficits, and increased anxiety. After intensive rehabilitation, the child was able to return to school with a modified class schedule and increased learning supports. This child, who meets diagnostic criteria for hypothalamic syndrome (1), continues to receive care from multiple specialty teams, including pediatric endocrinology, neuro-oncology, neurosurgery, neuro-ophthalmology, neuropsychology, psychiatry, hepatology, sleep medicine, occupational therapy, and physical therapy.

## Background, pathophysiology, and diagnostic criteria

**Figure 2:**
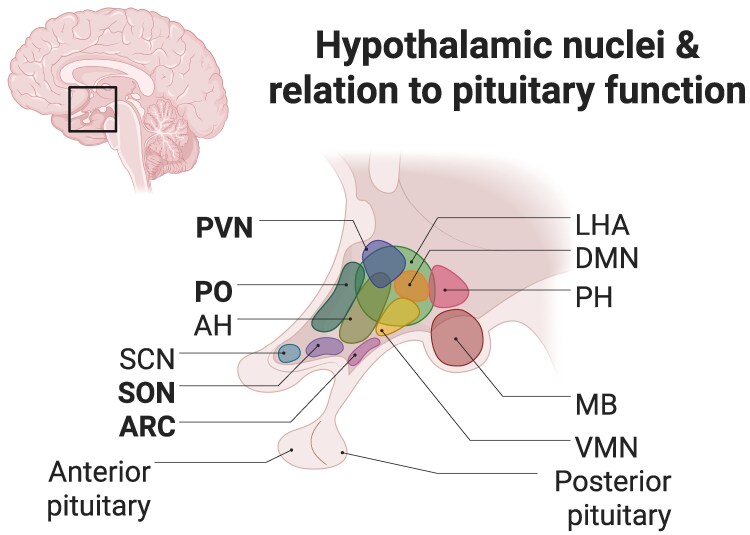
Key hypothalamic nuclei in the pathogenesis of hypothalamic syndrome. Injury from tumors and/or treatment to the nuclei in bold text disrupts the functions shown in detail in Table 1. Lateral hypothalamic area (LHA), suprachiasmatic nucleus (SCN), dorsomedial nucleus (DMN), ventromedial nucleus (VMN), anterior hypothalamic area (AH), posterior hypothalamic area (PH), and mamillary body (MB) contribute to feeding and metabolism (LHA, DMN), sleep and circadian rhythms (SCN, DMN, AH, PH), and memory (MB) (not comprehensive). Connections outside hypothalamus are also critical (not shown). AgRP, agouti-related protein; CCK, cholecystokinin; GLP1, glucagon-like peptide 1; POMC, proopiomelanocortin; PYY, peptide YY. Created in BioRender. Mccormack, S. (2026) https://BioRender.com/kqehxlz.

Many individuals with hypothalamic and pituitary tumors survive their tumors in the near term, only to develop health problems that limit well-being in the long term. Since the early 20th century, endocrinologists have recognized that hypothalamic tumors can cause not only multiple pituitary hormone deficiencies, but also a severe form of obesity, the co-occurrence of which was named Babinski-Fröhlich syndrome. Clinicians caring for such patients have also appreciated the presence of other highly morbid symptoms, including abnormal sleep and circadian function, temperature dysregulation, and behavior changes (2). The range of effects of hypothalamic injury follow from the essential roles of the hypothalamic nuclei, as illustrated in Fig. 2 and accompanying Table 1. Formal diagnostic criteria, including the common symptoms and a scoring system (1), were proposed to encompass both acquired and congenital forms of hypothalamic syndrome. These criteria include assessment of background risk from radiologic assessment of the hypothalamus and/or risk for a genetic disorder associated with hypothalamic syndrome, and then assessment of multiple clinical domains: status of pituitary hormone function, including AVP-D with or without thirst; appetite and weight trajectory; sleep and circadian function; temperature regulation; and behavior, in particular related to memory and impulse control. As previously summarized, craniopharyngioma is the prototypic suprasellar tumor leading to acquired hypothalamic syndrome, but other tumors (eg, low-grade gliomas, germinomas) also confer increased risk. Injury to the hypothalamus, whether from the tumors affecting this brain area or their treatment, leads to acquired hypothalamic syndrome in approximately 80% of individuals, depending in large part on the tumor characteristics (1, 2). Nononcologic processes, including traumatic brain injuries, can also cause acquired hypothalamic syndrome. A growing body of evidence supports the heterogeneity of this condition, with individuals experiencing many distinct and unique combinations of problems (3). Relevant to clinicians monitoring such patients, manifestations can also change over time.

**Table 1:** Hypothalamic nuclei and functions

Nucleus	Hormone(s)	Other roles
Preoptic nucleus	Gonadotropin-releasing hormone: puberty	Temperature sleep and circadian rhythm regulation
Paraventricular nucleus	Thyrotropin-releasing hormone: thyroid Corticotropin-releasing hormone: adrenal AVP: water balance OXT	Feeding and metabolism: GLP1 and leptin signaling Sympathetic tone
Arcuate nucleus	Growth hormone–releasing hormone: growth	Feeding and metabolism: POMC and AgRP signaling, insulin, ghrelin, leptin, PYY, CCK, GLP1 Sympathetic tone
Supraoptic nucleus	AVP: water balance OXT	Sleep and circadian: melatonin

Abbreviations: AgRP, agouti-related protein; AVP, vasopressin; CCK, cholecystokinin; GLP1, glucagon-like peptide 1; OXT, oxytocin; POMC, proopiomelanocortin; PYY, peptide YY.

A recently published review provides a detailed, comprehensive discussion of pathophysiology and management options (4). As shown in Fig. 2, the manifestations of hypothalamic syndrome follow directly from injury to hypothalamic nuclei. As an illustration, the preoptic nucleus produces gonadotropin-releasing hormone, and thus regulates puberty, so individuals with injury to this area can have hypogonadotropic hypogonadism requiring pharmacologic replacement. The preoptic nucleus also influences regulation of body temperature, sleep, and circadian rhythm; therefore, lesions affecting this region can result in temperature instability, insomnia, and circadian phase misalignment. Fig. 2 illustrates many of the known functions of the hypothalamus that can be affected; many other functions are less well characterized and/or are yet to be discovered, and may help to more fully explain the range of symptoms individuals experience. Hypothalamic syndrome represents a substantial burden for patients and families (5-8), and correspondingly, poses a considerable challenge to clinicians. The purpose of this piece is to serve as a brief, pragmatic guide to the multidisciplinary, collaborative evaluation and management of hypothalamic syndrome. For several of the manifestations of hypothalamic syndrome (eg, obesity), there are now evidence-supported therapies, whereas for others (eg, disordered temperature regulation), there are few studies demonstrating effective approaches. Regardless, increasing both patient/family and clinician awareness of hypothalamic syndrome is expected to lead to increased use of available interventions, including supportive measures, and to catalyze new investigations. We consider each dimension of hypothalamic syndrome separately, and conclude with a brief “checklist” that clinicians might consider using in preparing for visits with patients who are at risk for or have hypothalamic syndrome.

## Approach to management

### Radiographic assessment

A number of scoring systems have been proposed to predict the development of obesity and other symptoms following hypothalamic injury, mostly from brain tumors (9). The scoring system cited by the hypothalamic syndrome diagnostic criteria uses a 3-component scale: no hypothalamic involvement, anterior hypothalamic involvement only (sparing the mamillary bodies), or any posterior hypothalamic involvement (and/or the mamillary bodies), with posterior hypothalamus/mamillary body involvement having the worst prognosis (10). A separate system produces a composite score (0-7) based on the extent of the lesion in several key areas: the floor of the third ventricle (0-1), mamillary bodies (0-1), and anterior (0-1), medial (0-1), and posterior (0-1) hypothalamus, as well as the absence or presence of third ventriculomegaly (0-1) and lateral ventriculomegaly (0-1). The resulting score is predictive not only of weight gain (11), but also, in at least one study, response to antiobesity medication (12). Other features of imaging are being investigated for prognostic purposes, and may in the future inform efforts for prevention or early intervention for hypothalamic syndrome.

### Multiple pituitary hormone deficiencies

The hypothalamus controls corresponding pituitary hormone production by producing vasopressin (released by the posterior pituitary), corticotrophin-releasing hormone (for adrenocorticotropin), thyrotropin-releasing hormone (for thyrotropin), GH–releasing hormone (for GH), and gonadotropin-releasing hormone (for luteinizing hormone and follicle-stimulating hormone). Deficiencies of these hypothalamic and/or pituitary hormones result in central adrenal insufficiency (AI), GH deficiency (GHD), central hypothyroidism, and hypogonadotropic hypogonadism; in prepubertal age children, hypothalamic injury can also disrupt typical pubertal timing and precipitate early or precocious puberty (13).

Vasopressin is produced in the paraventricular and supraoptic nuclei of the hypothalamus and promotes retention of free water in response to hyperosmolality and hypotension. The hypothalamus also affects volume status by regulating thirst in response to these signals. In hypothalamic syndrome, disorders of water balance are common, variably including AVP-D, syndrome of inappropriate AVP secretion (either transient or permanent), reset osmostat, or absent thirst (adipsia). All require close monitoring of volume status, fluid intake, and blood sodium concentrations (14). Inadequately controlled AVP-D often results in nighttime awakenings to drink and/or urinate, which exacerbate fatigue in individuals who may already have disordered sleep. As a result, management of overnight symptoms is a priority. Additionally, individuals with intact thirst should be permitted to drink water to thirst. They should not be permitted ad libitum sweet beverages, though, since they are at risk to drink these to excess, which could precipitate hyponatremia if they are also taking a vasopressin analogue. Individuals with AVP-D may also be deficient in oxytocin, another hormone produced in the posterior hypothalamus. The potential role of oxytocin replacement to achieve benefits for metabolism, bone health, and psychological well-being is under active investigation (15). Oxytocin is not currently Food and Drug Administration approved for any of these indications in the United States, and more research is needed to establish the most appropriate formulation, dose, and indication(s), as well as to fully elucidate safety considerations.

Treatment of central AI is essential to prevent adrenal crisis. However, overtreatment can contribute to weight gain in individuals of all ages and poor linear growth in children. In adults, guidelines propose total daily hydrocortisone doses of 15 to 20 mg per day in 2 or 3 divided doses, with the highest dose in the morning, with a view to approximating typical circadian patterns; other formulations are also presented, typically for convenience and/or patient preference (16). Dose individualization is important. Much remains to be learned about daily glucocorticoid replacement, including amount, absorption, timing, and the use of an alternative (eg, longer-acting formulations) (17). In our practice, we typically begin with doses of 6 to 8 mg/m^2^/day divided twice daily, with approximately two-thirds in the morning and approximately one-third in the early afternoon for daily replacement; individuals with frequent adrenal crises and/or hypoglycemia on awakening may require higher and/or 3-times-daily dosing. Doses may be divided evenly for convenience or simplicity. Acute illness or procedures require “stress-dose” steroids to avoid adrenal crisis.

With respect to thyroid hormone, untreated central hypothyroidism can lead to fatigue, poor growth with ongoing weight gain, and dyslipidemia (18); thus, there is symptom overlap with other aspects of hypothalamic syndrome. It can thus be challenging to distinguish symptoms attributable to suboptimally treated central hypothyroidism to those related to other aspects of hypothalamic syndrome, such as impaired temperature regulation and disordered metabolism. We recommend treatment with levothyroxine with a goal of keeping thyroxine (T4)/free T4 in the upper half of the normal reference range (19-21). In primary hypothyroidism, the addition of twice-daily 3,5,3′-triiodothyronine (T3) replacement to levothyroxine is considered, especially if T3 levels are low, if individuals have persistent symptoms that could be related to hypothyroidism (22). One small case series appeared to demonstrate a benefit of T3 supplementation for weight loss (23), but this was not replicated (24). Thus, while such an approach could be considered in central hypothyroidism, combination therapy is not typically recommended (20). Thyrotropin levels are not reliable in patients with secondary hypothyroidism and should no longer be used to guide treatment after the diagnosis is made.

With respect to GH, children with untreated GHD have decreased linear growth, except sometimes when obesity is also present, so-called “growth without GH.” (25, 26) Individuals with untreated GHD often have impaired lipid profiles and increased visceral adiposity, and often report decreased energy and diminished quality of life. As a result, GH replacement is an important component of treating hypothalamic syndrome (27). With respect to the timing of GH initiation in deficient individuals, historically, a theoretical concern for risk of primary tumor recurrence or secondary neoplasia related to GH use led to caution, and delays of a year following treatment for some brain tumors (28). Ongoing accumulating evidence of the safety of GH replacement has led to more recent guidelines (29) and other reviews (30) suggesting initiation as early as 3 months post completion of therapy in individuals with craniopharyngioma. In all individuals, collaborative decision-making with the patient, family, and oncologist is essential, and especially so for those with, for example, chronic low-grade gliomas, who might have residual tumor.

With respect to sex steroid replacement, hypogonadism should be appropriately treated as in other individuals with hypogonadotropic hypogonadism, including pubertal induction for children, and consideration of fertility in those desiring it (20, 31). The timing of pubertal induction is individualized, with many individuals starting at typical ages (ie, 11 years in girls and 12 years in boys) (31), but some starting later if, for example, maximizing linear growth is a consideration in those with short stature who warrant additional time for growth to permit attainment of height potential. The many expected benefits of adequate replacement can be particularly helpful in hypothalamic syndrome, so sex steroid replacement should not be delayed too long. For example, girls with craniopharyngiomas have lower bone mineral density than controls, and this can be improved with timely initiation of estrogen; testosterone replacement in boys with hypogonadism increases muscle mass, which influences resting energy expenditure (32).

### Hypothalamic obesity

Hypothalamic obesity is a key feature of hypothalamic syndrome, and much has been learned about pathophysiology that has informed the development of therapies (33). Acquired hypothalamic obesity refers to the excess weight gain that is precipitated by physical injury to the hypothalamus, often by a tumor or its treatment. The hypothalamus regulates energy expenditure and eating behaviors; in general, the more extensive the hypothalamic injury, especially of the mamillary bodies and posterior hypothalamic area, the more likely excess adiposity will develop (34). Although some patients with hypothalamic obesity will endorse hyperphagia, others will gain weight even in the absence of changes in eating, a phenomenon attributable to a substantial decrease in metabolic rate (35). In one survey of 353 individuals with acquired hypothalamic dysfunction, obesity was the most frequently reported health problem, affecting 50.7%, followed by fatigue, reported by 48.2% (7), which underscores the importance of addressing this highly morbid complication.

Healthful nutrition and physical activity, as well as other aspects of lifestyle modification, are critical aspects of treatment, especially to prevent or mitigate obesity-related health problems, but are rarely sufficient to address weight gain; as a result, pharmacotherapy is playing an increasing role (36, 37). Individuals with acquired hypothalamic obesity often have both impaired muscle strength and low fitness (38), so exercise should be encouraged with consideration of comorbidities, although there are currently no evidence-supported recommendations for individuals with hypothalamic obesity. Public health guidelines typically recommend a combination of aerobic and resistance training that may initially be out of reach for individuals with hypothalamic obesity who are deconditioned. It may be helpful for individuals to work with an exercise physiologist and/or physical therapist to address specific problems, such as with muscle strength or balance, and thus gradually increase exercise tolerance.

It is challenging to conduct rigorous trials of nutrition interventions, especially in hypothalamic obesity; currently available data suggest that these are not typically very effective for weight loss (32). Even so, attention to nutrition is crucial. Our observation is that individuals who consume a diet with plenty of lean protein, healthful fat, vegetables and other high-fiber, complex carbohydrates, with limited amounts of simple and/or added sugars, are most successful. One speculation is that hypothalamic damage leads to increased parasympathetic signaling resulting in hyperinsulinemia, increased fat storage, and increased drive to eat (39). In the previously cited patient survey, 25.2% of respondents felt they had not received adequate nutritional advice when attempting to lose weight (7). Ideally, a dietitian would care for all individuals with hypothalamic syndrome in the context of a multidisciplinary clinic, but other creative solutions (eg, advice via telemedicine from dietitians in other locations) may be necessary to overcome local resource limitations.

Encouragingly, especially given the inadequate response to lifestyle modification, pharmacotherapies are being used for individuals with hypothalamic obesity. The advent of more efficacious, and more convenient, weekly glucagon-like peptide-1 receptor agonists (GLP1RAs) has led to their clinical use in hypothalamic obesity. The degree of weight loss in case series with earlier-generation GLP1RAs was quite variable (40), and one previous clinical trial of exenatide in hypothalamic obesity did not meet its prespecified primary end point of change in BMI, but did lead to a discernible loss of fat mass relative to placebo (41). In contrast, in 2024, a case series demonstrated that 25 of 26 individuals with hypothalamic obesity lost weight in response to semaglutide, with a mean weight loss of 13.4 kg after 12 months (42). A multicenter, retrospective study of more than 100 individuals treated with GLP1RAs also demonstrated that weight loss is achievable, and highlights important safety considerations regarding using these medications in individuals with concomitant AVP-D and AI (43). Fewer data are available in children with hypothalamic obesity, even though GLP1RAs are approved for obesity in the United States for children as young as 12 years, and as young as 10 years for other indications. We have observed weight loss using weekly GLP1RAs in children with hypothalamic obesity. However, as in adults, access remains challenging, especially for children, for whom there are fewer currently approved other indications (eg, metabolic-associated liver disease, obstructive sleep apnea) compared to adults. If trialing a GLP1RA, we recommend starting at the lowest dose, and titrating the dose slowly based on weight loss and appetite. If an individual is losing weight but continues to have substantial hyperphagia, we recommend increasing the dose to address their hyperphagia. Additionally, dosing must be individualized to address adverse effects, which can be more severe in individuals with multiple pituitary hormone deficiencies who must take oral medications and maintain hydration. For individuals who do not experience adequate weight loss on lower doses of GLP1RAs, but develop intolerable gastrointestinal side effects on the next available higher increment, intermediate dosing of semaglutide using specific pens that can dial incremental amounts may be an option (44).

Options also exist beyond GLP1RAs, specifically developed and tested in hypothalamic obesity. Setmelanotide, a melanocortin-4 receptor (MC4R agonist), has been approved for use in specific monogenic forms of hypothalamic obesity, including related to pathogenic variants in *POMC*, *PCSK1*, and *LEPR*, as well as Bardet-Biedl syndrome. In a phase 3 study of setmelanotide in individuals with acquired hypothalamic obesity, after 52 weeks, participants had a mean change in BMI of −16.5% with setmelanotide vs +3.3% in the placebo group (45). Regulatory approvals are pending for this indication. As when used for other forms of hypothalamic obesity, the most common side effects were hyperpigmentation, nausea, and vomiting. Setmelanotide causes hyperpigmentation because of off-target cross-reactivity at the melanocortin 1 receptor in melanocytes. Hyperpigmentation typically appears within a few weeks of treatment initiation. Preexisting nevi can become darker, and new melanocytic nevi may appear; thus, regular dermatologic monitoring is appropriate. Hyperpigmentation is expected to reach a plateau, and fade if medication is discontinued. Nausea and vomiting also typically appear soon after treatment initiation; more gradual uptitration can help with tolerability. On balance, the favorable risk-benefit balance of this medication thus holds promise for treatment of acquired hypothalamic obesity.

In our practice, we consider GLP1RAs and MC4RAs (once available), either alone or in combination, for eligible individuals with hypothalamic obesity needing pharmacotherapy. The optimal initial strategy remains the focus of investigation. If these medications are not available and/or not well tolerated even with slow, careful titration, other options should be considered.

Some affected individuals with hyperphagia or their caregivers will ask about extended-release diazoxide, which was approved in 2025 to treat hyperphagia in patients with Prader-Willi syndrome (PWS). In PWS, extended-release diazoxide improved severe hyperphagia only (46) and while body fat mass remained stable, lean body mass increased (47). Similar to a previously performed study in individuals with hypothalamic pituitary lesions (48), diazoxide use was associated with hyperglycemia. A separate, 6-month, open-label pilot study in individuals with hypothalamic obesity from craniopharyngioma (n = 9) combined diazoxide with metformin to avoid hyperglycemia led to attenuated weight gain relative to pre treatment (39). Based on the experience to date, and the risk for side effects, which extend beyond hyperglycemia to fluid retention, and modest, if any, anticipated benefit for weight loss, we do not recommend using diazoxide for acquire hypothalamic obesity outside the research setting.

A number of other agents have been used clinically for hypothalamic obesity. One ongoing trial is testing combined phentermine and topiramate in individuals with hypothalamic obesity (NCT06299891); empiric data are limited, but this oral agent already approved in the United States for adolescents age 12 years and older with obesity could be considered with careful individualization of the risk-benefit profile, including attention to potential cardiac effects of stimulants, and electrolyte changes and kidney stone risk with topiramate.

With respect to another alternative, metformin, in one small series, metformin helped to stabilize weight in individuals already receiving an intense lifestyle modification intervention (49). While not a first-line choice to achieve weight loss in hypothalamic obesity, based on minimal anticipated benefit, metformin may be useful to address insulin resistance or type 2 diabetes mellitus, and is inexpensive, so may be an option for individuals for whom GLP1RAs, for example, are not a choice.

Stimulants should be strongly considered in individuals with excess appetite who also have other indications for stimulants such as inattention, attention deficit hyperactive disorder (ADHD), and/or excess daytime fatigue, as stimulants can help address excess weight gain while also improving attention and energy. In a retrospective study, dextroamphetamine (50) has demonstrated favorable benefits with respect to weight loss (or attenuation of weight gain) while also achieving decreases in hyperphagia and improved energy (50). In another retrospective study, methylphenidate (51) also had modest effects. Ephedrine, caffeine, and phentermine alone or in combination with topiramate have also been proposed (36, 37) Finally, a small trial using tesofensine plus metoprolol (the latter to avoid potential adverse cardiovascular effects of tesofensine) resulted in weight loss in hypothalamic obesity (52), but at the time of this writing, tesofensine is not approved for clinical use.

All individuals with hypothalamic obesity should be counseled with respect to the range of options available to manage excess weight gain, also considering the implications for any other complications of hypothalamic syndrome present. Shared decision-making is essential because every individual has unique priorities and circumstances, and our evidence is incomplete. We need to ask about individuals’ successes and struggles, so we can celebrate achievements and iterate to overcome barriers. Individuals and their families invest a great deal of time and energy into managing their condition, effort that is not always reflected in the measurement on the scale, but is vital to acknowledge and support.

### Cardiovascular manifestations

Although not part of the diagnostic criteria for hypothalamic syndrome per se, the many cardiovascular manifestations of hypothalamic injury contribute both to morbidity and excess mortality in affected individuals. First, the hypothalamus, in particular the ventromedial and dorsomedial hypothalamic nuclei, integrates signals from the autonomic nervous system that govern physiologic processes including heart rate, blood pressure, respiratory rate, body temperature, and digestion (53-56). Decreased heart rate variability, the loss of the typical beat-to-beat fluctuation in rhythm, is a manifestation of autonomic impairment that has been demonstrated in individuals with hypothalamic injury (57, 58). The autonomic nervous system also supports the cardiovascular response to exercise. Autonomic impairment may contribute to the fatigue, exercise intolerance, and decreased voluntary physical activity reported by many individuals with hypothalamic syndrome (35, 59, 60).

The autonomic nervous system is also essential in maintaining hemodynamic stability in response to stresses such as illness, injuries, or operative procedures. In individuals with hypothalamic syndrome with multiple pituitary hormone deficiencies (AVP-D, AI) that confer vulnerability to dehydration and hypotension, autonomic impairment is a further risk factor for possible life-threatening complications from stresses (61). Along with electrolyte abnormalities related to hormone deficiencies and concurrent use of high-risk medications, autonomic impairment might further exacerbate risk for QTc interval prolongation that has been noted in some individuals with hypothalamic/pituitary tumors (62, 63).

Childhood survivors of craniopharyngioma (64), the prototypic tumor causing hypothalamic syndrome, have elevated cardiovascular disease risk factors that portend future excess rates of premature mortality (65). Adverse cardiac remodeling is evident even in the youngest survivors, and worsens with increasing age and by adulthood, also with obesity (66).

### Fatigue, sleep, and circadian disorders

Sleep is critical for overall health. Insufficient and/or nonrestorative sleep can have profound effects on multiple dimensions of health (metabolic, cardiovascular, immune function) and well-being (mood, learning). Excessive daytime sleepiness from narcolepsy, sleep-disordered breathing, and/or circadian rhythm dysregulation has been reported in from 25% to 100% of individuals with craniopharyngioma, depending on the cohort and tools used to assess (67). Consensus exists that excess daytime sleepiness is likely underreported, in part because of underrecognition even among affected individuals and their families. For example, one study screened children with craniopharyngioma using multiple sleep latency testing, and 82% met these objective criteria for excess daytime sleepiness, while only 29% endorsed this symptom on a validated questionnaire, the modified Epworth Sleepiness Scale (68).

Sleep disruption is expected in hypothalamic syndrome because the hypothalamus plays a critical role in maintaining healthful sleep-wake cycles. As an illustration, type 1 narcolepsy, which includes cataplexy in addition to excess sleepiness, is caused by the destruction of the hypothalamic hypocretin neurons that promote wakefulness. (Cataplexy refers to the episodic, sudden loss of muscle tone lasting from several seconds up to 2 minutes triggered by strong emotions, during which the individual remains awake and aware.) The etiology of type 2 narcolepsy (sleepiness without cataplexy) is less clear but is also associated with hypothalamic injury (67). If the suprachiasmatic nucleus of the hypothalamus that regulates circadian function is injured, individuals with lesions such as craniopharyngioma may experience disordered circadian rhythm, as manifested by disruptions in the nighttime sleep-promoting hormone melatonin (69). Untreated sleep-disordered breathing, which is more common but not exclusively seen in individuals with obesity, can also contribute to excess daytime sleepiness. Some, though not all, studies suggest that individuals with craniopharyngiomas have higher rates of sleep apnea when compared to weight-matched controls (70, 71); thus, it is possible that hypothalamic injury predisposes to apnea even independent of injury-related obesity.

Fatigue is a commonly reported symptom that deserves particular attention. While this symptom is incompletely understood, and individuals mean different things when they report fatigue, multiple known factors could plausibly contribute to fatigue in individuals with hypothalamic syndrome. Commonly reported fatigue (72, 73) could potentially be related to disordered signaling from γ-aminobutyric acid (GABA)-ergic neurons in the suprachiasmatic, ventrolateral, and preoptic nuclei (74, 75). As noted previously, inadequately treated pituitary hormone deficiencies can also result in fatigue, so ensuring adequate replacement of pituitary hormones is critical to the multicomponent treatment of fatigue.

Pharmacologic treatments continue to evolve. In one small study (n = 10 adults with hypothalamic obesity following treatment for craniopharyngioma), 6 mg of melatonin 30 minutes prior to bed improved daytime sleepiness (76). Methylphenidate, dextroamphetamine, and modafinil and combinations of modafinil and dextroamphetamine have been used to treat narcolepsy and excessive sleepiness; practice parameters for their use have been published (4, 77). Although continuous positive airway pressure treatment has not consistently been shown to improve excess daytime sleepiness in those with sleep-disordered breathing (67), its use remains strongly recommended because it avoids frequent apneic episodes and potential sequelae of untreated obstructive sleep apnea, including pulmonary hypertension. Treatment of fatigue generally requires multiple interventions and often remains a challenge, but this highly morbid symptom remains an important and patient-relevant concern to address.

### Temperature dysregulation

The hypothalamus has a critical role in maintaining body temperature via circulating humoral signals (eg, thyroid hormone) and regulation of nervous system inputs (eg, sympathetic tone) (78). Warm-sensitive neurons in the preoptic area of the hypothalamus integrate temperature information from the periphery and control some of the many signals that affect thermogenesis (79). These are the same neurons that produce fever in response to inflammatory cytokines in the context of infection. Individuals generate heat at rest while eating and digesting (the “thermic effect of food”), while being active and exercising, and also as an adaptive response to changes in environmental temperature. Shivering in response to cold exposure, an important cold-induced thermogenic response, is also governed by the hypothalamus and its modulation of the sympathetic nervous system. Peripheral vasodilation in response to heat, a critical mechanism to dissipate heat, is also influenced by the same brain areas. Therefore, hypothalamic injury can lead to temperature extremes and associated systemic effects, that is, hypothermia (80) and hyperthermia (81). The tendency to experience temperature fluctuations is part of the diagnostic criteria for hypothalamic syndrome, with more severe excursions making the diagnosis more likely. As with other complications of hypothalamic syndrome, hypothalamus-sparing tumor treatment approaches tend to preserve more capacity for temperature regulation. Distinguishing temperature fluctuations arising solely from hypothalamic injury vs from more serious causes such as serious infection can be challenging. Supportive measures, including avoiding exposure to extremes in temperature, and ensuring appropriate pituitary hormone replacement, can be helpful, but in some individuals it is difficult to avoid these manifestations entirely. For persistently elevated temperature, strategies from individuals with traumatic brain injury, including administration of chlorpromazine, may be effective, although there is not substantial literature specifically in hypothalamic syndrome (81).

### Behavioral disorders

Behavioral problems are common in individuals with hypothalamic syndrome. In the proposed diagnostic criteria, behavioral problems are defined as, “the presence of obsessive compulsive symptoms (defined as fixation on certain topics, repetitive behavior or rituals, difficulty with changes), hoarding (collects items not needed), rage.” (1) Much of the related literature originates from studies in individuals with PWS, so not all phenomena necessarily translate to acquired hypothalamic syndrome. Some individuals with brain tumors develop obsessive compulsive symptoms, but these manifestations seem infrequent enough that they were described in a case report (82). Hoarding, unrelated to food-seeking, is also infrequently reported (83). Systematic, prospective data collection would give more definitive estimates of behavior problems. Food-seeking behaviors have been studied more comprehensively. Individuals with hyperphagia spend more time “talking or engaging with food” than those without hyperphagia, and food interferes with daily functioning (84). The effect of these hyperphagia behaviors is decreased quality of life for affected individuals and increased burden for caretakers; obesity without hyperphagia does not have the same effects (84). Interestingly, when assessed using validated eating behavior questionnaires, individuals with craniopharyngiomas did not endorse more pathological eating behaviors compared to BMI-matched controls, suggesting either a heterogeneity of causes, lack of insight, and/or highlighting the prevalence of problems even in otherwise health controls with increased BMI (85).

The limbic system affects emotional regulation and is integrally connected to the hypothalamus (86), so when the connection between the two is impaired, emotional lability can occur. The limbic system has also been implicated in the development of ADHD, which may explain symptoms of inattention and difficulty focusing that may develop in individuals with hypothalamic syndrome. However, other causes, such as disturbed sleep, also need to be considered.

Cognitive impairment is also reported in individuals with hypothalamic syndrome. Individuals with craniopharyngioma have impaired episodic memory and slowed processing speeds that are associated with changes in hippocampal volumes and white matter alterations on brain imaging (87). The larger the extent of hypothalamic involvement, the worse individuals perform on tasks related to executive function. These deficits translate into impaired functional capabilities relative to healthy control cohorts, and portend worse academic performance and lower quality of life. Even with these considerations, on average, IQ testing results are not substantially decreased from average; thus, substantial potential remains for learning with appropriate supports (88).

Peer relationships also pose a challenge for many individuals with hypothalamic syndrome; those with obesity fare even worse than those without, and more than is accounted for by weight gain alone (5). Some investigators have speculated that deficiency of the prosocial hormone oxytocin in individuals with hypothalamic syndrome could explain some of the difficulties with peer relationships (89). As noted earlier, investigations are ongoing as to the potential therapeutic relevance of oxytocin replacement.

The multiplicity, complexity, and variability of neurobehavioral changes in individuals with hypothalamic syndrome mean that there is no simple, one-size-fits-all solution. A coordinated approach including the individual and family, primary care physician, endocrinologist, psychologist, psychiatrist, sleep clinician, and the school team is warranted, but often challenging to organize. At-risk individuals should be formally evaluated for neurocognitive and/or learning deficits, and children should have school accommodations to address any cognitive and behavioral problems. Appropriate treatment of pituitary hormone deficiencies, sleep disorders, and ADHD are essential for better focus. Acknowledgement of substantial ongoing psychosocial stresses related to the diagnosis and sequelae is also vital. Addressing caregiver burden is another important goal (8).

## Back to the case

**Figure 3:**
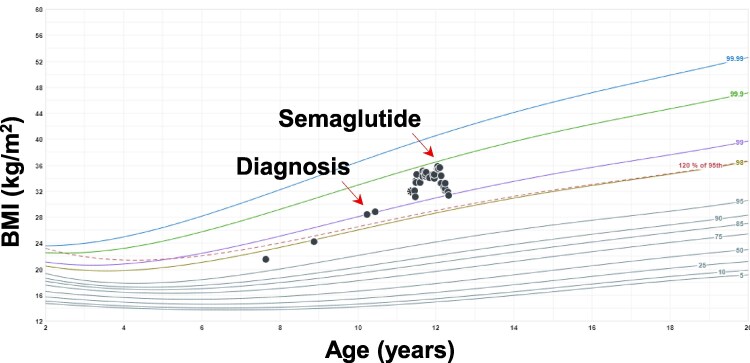
Body mass index (BMI) trajectory of the case described, at diagnosis and after initiation of semaglutide, as shown by the arrows. BMI already exceeded the 95th percentile at diagnosis, and quickly increased to a peak of 148% of the 95th percentile within 9 months. After the patient turned age 12 years, approval for a glucagon-like peptide 1 agonist (semaglutide) for obesity was obtained, with a decrease to 124% of the 95th percentile over the first approximately 6 months of treatment.

The child continued to undergo MRI surveillance at intervals and was without tumor recurrence. The child continued to receive pituitary hormone replacement; GH was initiated by 6 months post resection to address both decreased linear growth as well as increased visceral adiposity and metabolic-associated steatotic liver disease. A fluid target, adjustable based on contingencies (eg, excess breakthrough) was initiated for AVP-D without intact thirst. Emergency letters were updated at each visit. Once the child turned 12 years, semaglutide was obtained for the obesity indication, and led to some initial weight loss, including a 14% decrease in BMI, representing a decrease from 148% of the 95th percentile BMI to 124% of the 95th percentile BMI (Fig. 3). Dose was uptitrated very slowly, and medication needed to be paused intermittently for both symptoms (excess nausea and vomiting and associated hypernatremia) as well as intercurrent illnesses (eg, gastroenteritis). Liver function tests varied, but overall alanine transaminase decreased from a peak of 3 to 4 times the upper limit of the reference range to approximately 2 times the upper limit of the reference range; prior to initiation of semaglutide, magnetic resonance elastography showed steatosis, with fat fraction between 12% and 14%, and increased liver stiffness, reflecting congestion and/or fibrosis, at 4.8 kPa. Diagnostic biopsy was declined, and a presumptive diagnosis of metabolic-associated steatotic liver disease was made. Follow-up imaging after semaglutide is pending. Continuous positive airway pressure was initiated for obstructive sleep apnea. Supportive measures at home and school were taken to avoid exposure to temperature extremes, including during physical activity. Physical and occupational therapy continued. A selective serotonin reuptake inhibitor was initiated for anxiety with good effect. Neuropsychological evaluation guided social-emotional and cognitive learning supports, and social work facilitated an application for disability benefits.

## Conclusion

Hypothalamic syndrome is a complex condition that requires a multidisciplinary treatment approach with individualized care. A brief “checklist” for evaluation and management of hypothalamic syndrome (Table 2) is not intended to be comprehensive, but instead can help prompt the clinician in preparing for visits. Management should address each component individually and consider their interaction. A patient-oriented, multidisciplinary approach to treatment is most likely to address the most relevant concerns. Because this population is relatively small, all stakeholders, including individuals and families, clinicians, and investigators, along with pharmaceutical colleagues, regulators, and payers, will need to collaborate to develop and implement effective treatments for acquired hypothalamic syndrome. Even more, efforts to prevent or reverse injury to the hypothalamus in the course of tumor treatment (90) could help prevent the onset of this highly impactful condition.

**Table 2:** Brief clinical visit checklist for acquired hypothalamic syndrome

Domain	Assessment item
Risk assessment	Review brain MRI for evidence of hypothalamic injury, with particular attention to mamillary bodies and posterior hypothalamic area
	Confirm tumor surveillance is current (oncology patients)
	Ensure neuro-ophthalmology surveillance is current (acuity, visual fields)
Pituitary hormone replacement	Confirm emergency letter is updated
	AVP deficiency: document thirst status (intact vs absent); assess for symptoms of polyuria or polydipsia; update desmopressin doses and fluid targets
	Thyroid: target free T4 in upper half of reference range
	Adrenal: use lowest effective daily glucocorticoid dose to avoid symptoms; ensure stress doses available and teaching is current
	Growth hormone: consider early replacement if deficient
	Sex steroids: ensure appropriate for age and pubertal stage
Adiposity and eating behavior	Review BMI trajectory
	Assess any hyperphagia symptoms (eg, food-seeking behavior, response to satiety cues)
	Discuss any environmental modifications taken to limit excess intake at home or school (eg, controlling access, limiting food rewards)
	Screen for sequelae of excess adiposity if present (blood pressure, lipids, glucose metabolism, liver disease)
	Consider pharmacotherapy if indicated for sequelae, rapid gain, and/or impairment in day-to-day activities
Sleep	Inquire about fatigue, snoring, excessive daytime sleepiness, insomnia
	Low threshold for polysomnography referral, even in absence of reported symptoms
Temperature regulation	Inquire about heat or cold intolerance (especially outdoors and with activity)
	Review supportive measures (timing of activity, appropriate clothing, hydration, cooling strategies and shade in warmer weather)
Behavior and cognition	Inquire about behavioral challenges including impulsivity, mood fluctuations
	Assess any learning difficulties and school or work functioning
	Inquire about peer relationships
	Low threshold for neuropsychology and/or psychiatry referral
Energy and activity	Assess fatigue and energy level
	Discuss physical activity; consider physical therapy or adaptive exercise program
Psychosocial support	Offer social work and community supports for individual and caregivers
	Assess quality of life; discuss transition planning if adolescent

Abbreviations: AVP, arginine vasopressin; BMI, body mass index; MRI, magnetic resonance imaging; T4, thyroxine.

## Data Availability

No new data were generated or analyzed in support of this research.

## Abbreviations

ADHD: attention deficit hyperactive disorder
AI: adrenal insufficiency
AVP: arginine vasopressin
AVP-D: arginine vasopressin deficiency
BMI: body mass index
GH: growth hormone
GHD: growth hormone deficiency
GLP-1: glucagon-like peptide-1
GLP1RAs: glucagon-like peptide-1 receptor agonists
MC4R: melanocortin-4 receptor
MRI: magnetic resonance imaging
PWS: Prader-Willi syndrome
T3: 3,5,3′-triiodothyronine
T4: thyroxine
